# Complementary and alternative medicine use among US Navy and Marine Corps personnel

**DOI:** 10.1186/1472-6882-7-16

**Published:** 2007-05-16

**Authors:** Tyler C Smith, Margaret AK Ryan, Besa Smith, Robert J Reed, James R Riddle, Gia R Gumbs, Gregory C Gray

**Affiliations:** 1 Department of Defense Center for Deployment Health Research at the Naval Health Research Center, San Diego, CA, USA; 2 Air Force Research Laboratory, Wright-Patterson Air Force Base, OH, USA; 3 College of Public Health, University of Iowa, Iowa City, IA, USA

## Abstract

**Background:**

Recently, numerous studies have revealed an increase in complementary and alternative medicine (CAM) use in US civilian populations. In contrast, few studies have examined CAM use within military populations, which have ready access to conventional medicine. Currently, the prevalence and impact of CAM use in US military populations remains unknown.

**Methods:**

To investigate CAM use in US Navy and Marine Corps personnel, the authors surveyed a stratified random sample of 5,000 active duty and Reserve/National Guard members between December 2000 and July 2002. Chi-square tests and multivariable logistic regression were used to assess univariate associations and adjusted odds of CAM use in this population.

**Results and discussion:**

Of 3,683 service members contacted, 1,446 (39.3%) returned a questionnaire and 1,305 gave complete demographic and survey data suitable for study. Among respondents, more than 37% reported using at least one CAM therapy during the past year. Herbal therapies were among the most commonly reported (15.9%). Most respondents (69.8%) reported their health as being very good or excellent. Modeling revealed that CAM use was most common among personnel who were women, white, and officers. Higher levels of recent physical pain and lower levels of satisfaction with conventional medical care were significantly associated with increased odds of reporting CAM use.

**Conclusion:**

These data suggest that CAM use is prevalent in the US military and consistent with patterns in other US civilian populations. Because there is much to be learned about CAM use along with allopathic therapy, US military medical professionals should record CAM therapies when collecting medical history data.

## Background

In recent years, complementary and alternative medicine (CAM) has shown an increasing presence in the US health care system as well as in Canada, Australia, Denmark, and the United Kingdom, where reports of CAM use vary from 9 to 65% [1-10]. Over 40% of one US adult population sample reported they used at least one alternative therapy in 1997, an increase of 25% over the estimated CAM use in 1990 [1]. Furthermore, the number of visits to practitioners of alternative therapies and total expenditures related to alternative therapies also increased, rivaling or exceeding visits and expenditures for conventional medical care [1]. Reported trends for increasing CAM use within US adult populations underscore the importance of understanding CAM treatments and for including CAM in discussions of US healthcare policy and practice [11-15].

Characteristics of individuals choosing to use CAM and physician awareness of CAM use among US adult patients have been documented [1,12]. Reports suggest that people more likely to use some form of CAM are women ages 25–49 years, White, educated, and middle economic class [1,11,12,15-18]. Among the most commonly reported CAM therapies in civilian populations are chiropractic care, herbal medicine, relaxation therapy, and massage [1,11,12,15-18].

Concerns over the risks and benefits of unmanaged therapies [19-21] fuel interest and debate over the use of CAM in healthy US military populations. There is some fear that diet and training regimens of military personnel could be compromised by intake of unconventional supplements and use of unregulated practices that may result in unforeseen health consequences [22]. A recent study examined CAM use within a population of military families and reported that 28.2% used some form of CAM [23]. While the prevalence reported in the study was lower than that found in civilian studies, population characteristics and communication of CAM use to family physicians were consistent in the two populations. Other studies of US military veterans found that between 27.3% and 49.6% self-reported using some form of CAM [14,24]. However, CAM use in healthy, active military populations remains unclear. In the current study, we document the prevalence of CAM use and the characteristics of those who report CAM use in an active duty and Reserve/Guard US military population.

## Methods

### Study population

In December 2000, a random sample of 5,000 active duty and Reserve/Guard personnel was drawn from Navy and Marine Corps rosters of approximately 550,000. To ensure adequate power to make inferences among female service members, women were slightly oversampled to account for 20% of the invited participants. Demographic data for the 5,000 invited participants were provided by the Defense Manpower Data Center, Monterey Bay, California (DMDC). These data included age (categorized by approximate quartile age groups: 18 to 22 years, 23 to 26 years, 27 to 34 years, and 35 to 57 years), marital status, gender, race/ethnicity (White, Black, Hispanic, Asian/Pacific Islander, and other), service branch (Navy, Marine), service component (active duty or Reserve/Guard), Department of Defense (DoD) primary occupational specialty (10 major categories, defined by the DoD Occupational Conversion Index) [25], rank (enlisted or officer), highest level of education, and length of service.

This research has been conducted in compliance with all applicable federal regulations governing the protection of human subjects in research (Protocol NHRC.2001.0001).

#### Postal survey

The choice of questions and question layouts for the survey instrument were modeled after the questionnaire used by Astin in a study of CAM use in a civilian population [11]. To determine participants' self-perceived health status and conventional care utilization and satisfaction, the survey asked about general health, number of sick days in the last 12 months, degree of body pain in the last 4 weeks, frequency and type of conventional medical care sought, satisfaction and trust of medical care, and the prevalence of specific health problems within the past 12 months. The optically scanned 10-page survey instrument was designed to take approximately 30 minutes to complete.

Participants' use of CAM and other treatments was assessed through questioning participants about their use of practitioner-assisted CAM, self-administered CAM, and use of dietary supplements and diet programs within the past 12 months. Additionally, participants were asked to provide demographic data including race/ethnicity, level of education, military duty occupation, household income, marital status, and gender. In the event that self-reported demographic data were incomplete or missing, data acquired from DMDC were substituted. Prior to mailing the survey, a focus group consisting of active-duty Navy personnel reviewed the cover letter, consent form, and survey content and layout. Based on the focus group's comments and suggestions, the survey instrument was refined before the initial mailing. Additionally, a random sample of 33% of individuals who completed the initial questionnaire was sent a second survey consisting of a subset of questions from the original survey. These data were used to measure agreement with the participant's initial survey responses.

For all invited participants, both duty and home addresses were obtained from DMDC. A mailing schedule was followed based on a modified version of the Dillman protocol in which each invited participant could be mailed up to three surveys along with prepaid return envelopes [26]. The voluntary nature of participation and the confidentiality with which their data would be treated were noted and stressed in each mailing.

### CAM definition

We defined an individual as using CAM after self-reported use of one or more of the following treatments within the past year: acupuncture, biofeedback, chiropractic care, energy healing, folk remedies, herbal therapies, high-dose megavitamins, homeopathy, hypnosis, massage, relaxation, or spiritual healing. Though this CAM definition is similar to those used in several previous CAM studies, these analyses exclude treatments such as exercise, lifestyle diets, self-help groups, and psychotherapy because the distinction between CAM and conventional medicine is often less clear for these treatments [1,11,15,16]. While some information is lost when aggregating into CAM use versus non-CAM use, this allowed for comparison to other reports.

### Statistical analyses

After descriptive investigation of population characteristics, biivariate analyses with chi-square tests of association were performed to assess significant associations between CAM use and demographic or self-reported health questions. An exploratory logistic regression analysis was conducted to further assess significant associations and possible confounding while simultaneously adjusting for all other covariates in the model. From these analyses, a set of variables with *p *value significance characterized by values of 0.10 or less was retained for subsequent modeling. Multicollinearity among variables was investigated. Additionally, multiplicative interaction was investigated by introducing cross-product terms into the model to test for significance of interaction. The saturated logistic regression model was reduced by manual backward stepwise elimination, removing those variables that were not significant (as characterized by *p *values > 0.05) and that did not confound the other measures of association.

Additionally, to investigate reliability of questionnaire answers using a test-retest approach, Kappa statistics were used to determine the degree of non-random agreement [27], Cut-points for the agreement levels were as follows: Kappa = 0.8 to 1.0 to distinguish almost perfect agreement, 0.6 to 0.8 to distinguish substantial agreement, 0.4 to 0.6 to distinguish moderate agreement, 0.2 to 0.4 to distinguish fair agreement, and 0.0 to 0.2 to distinguish slight or poor agreement [28].

All analyses were conducted using SAS Version 9.1 software for Windows (SAS Institute Inc., Cary, NC) [29].

## Results

Among the 5,000 Navy and Marine Corps personnel identified through random sampling procedures, 3,683 were eligible to participate. Among the ineligible subjects, 16 did not meet the initial enrollment criteria and 1,301 could not be located. Of the 3,683 eligible subjects, 49 refused to participate, 2,188 did not respond after repeated mailings, and 1,446 responded. This resulted in a response rate of 39.3%. Respondents were slightly different than the targeted population of Navy and Marine Corps personnel, tending to be older, married, from officer ranks, in the Navy, and working in the field of health care (Table 1).

**Table 1:** Characteristics of Complementary and Alternative Medicine Survey Responders Compared With the Target Population

Characteristic	Target Population (n = 5,000)		Responders (n = 1,446)	
	Number	%	Number	%
Gender				
Male	4,000	80.0	1,121	77.5
Female	1,000	20.0	325	22.5
Age (years)				
18–24	1,982	39.6	327	22.6
25–31	1,391	27.8	396	27.4
32–38	922	18.5	377	26.0
39–57	703	14.1	346	24.0
Missing	2	<0.01	0	0
Marital status				
Unmarried	2,444	48.9	533	36.9
Married	2,348	46.9	819	56.6
Missing	208	4.2	94	6.5
Rank				
Enlisted	4,342	86.8	1,128	78.0
Officer	658	13.2	318	22.0
Service branch				
Navy	3,291	65.8	1,061	73.4
Navy, Reserve/Guard	228	4.6	74	5.1
Marines	1,481	29.6	311	21.5
Occupational category				
Combat specialists	1,071	21.4	302	20.9
Mechanical repair	929	18.6	244	16.9
Administrative	738	14.8	234	16.2
Electrical repair	620	12.4	203	14.0
Communications and intelligence	365	7.3	98	6.8
Health care	358	7.2	135	9.3
Supply	325	6.5	82	5.7
Craft workers	146	2.9	35	2.4
Technical	90	1.8	29	2.0
Students/trainees	89	1.8	23	1.6
Missing	269	5.4	61	4.2

For this analysis, complete demographic and survey data were available for 1,305 of the 1,446 personnel who had elected to participate and submitted their questionnaire (90.2%). The study population consisted of 77% men, 50% younger than 31 years, 59% married, 77% enlisted, 73% with some high school or college education, 67% White, and 68% earning less than $50,000 per year. Regarding health, nearly 70% reported very good or excellent health, 52% reported at least one sick day in the past year, 59% reported no or very mild body pain, fewer than half were very satisfied with their physician, more than half had complete trust in their physician, 99% reported no addiction to drugs or alcohol in the past year, and 95% reported that they were not obese (Table 2). In bivariate analyses, age, salary, general health, trust in their physician, addiction to alcohol or drugs, and obesity were not found to be significantly associated with CAM use (α = 0.10) and were excluded from further analyses.

**Table 2:** Characteristics and Unadjusted Associations of Complementary and Alternative Medicine (CAM) Use

Variable	Study Population n = 1,305		CAM Use n = 485 (37.2%)		*p *value^†^
	Number	%	Number	%*	
Gender					<0.01
Male	1,009	77.3	338	33.5	
Female	296	22.7	147	49.7	
Age (years)					0.99
18–24	300	23.0	114	38.0	
25–31	359	27.5	133	37.1	
32–38	345	26.4	126	36.5	
39–57	301	23.1	112	37.2	
Marital status					0.04
Unmarried	531	40.7	215	40.5	
Married	774	59.3	270	34.9	
Rank					<0.01
Enlisted	1,009	77.3	356	35.3	
Officer	296	22.7	129	43.6	
Service branch					<0.01
Navy	949	72.7	361	38.0	
Navy, Reserve/Guard	72	5.5	37	30.6	
Marines	284	21.8	87	51.4	
Occupational category					0.06
Combat specialists	285	21.8	107	37.5	
Mechanical repair	216	16.6	72	35.3	
Administrative	220	16.9	79	45.6	
Electrical repair	184	14.1	65	38.3	
Communications and intelligence	90	6.9	41	65.2	
Health care	128	9.8	49	35.9	
Supply	78	6.0	22	33.3	
Craft workers	33	2.5	13	39.4	
Technical	23	1.8	15	28.2	
Students/trainees	48	3.7	22	45.8	
Education level					0.02
Some high school or diploma	339	26.0	105	31.0	
Some college	611	46.8	235	38.5	
College degree or higher	355	27.2	145	40.9	
Race/ethnicity					<0.01
White	871	66.7	355	40.8	
Black	183	14.0	55	24.4	
Hispanic	119	9.1	29	30.1	
Asian/Pacific Islander	84	6.4	28	33.3	
Other	48	3.7	18	37.5	
Salary					0.49
<$19,999	245	18.8	90	36.7	
$20,000–$34,999	365	28.0	129	35.3	
$35,000–$49,999	275	21.1	96	34.9	
$50,000–$74,999	249	19.1	98	39.4	
≥$75,000	171	13.1	72	42.1	
General health					0.19
Very good or excellent	911	69.8	327	46.6	
Good	336	25.8	131	39.0	
Poor or fair	58	4.4	27	35.9	
Number of days spent in bed due to illness or injury in past year (sick days)					<0.01
None	622	47.7	203	32.6	
1–2	435	33.3	165	37.9	
3–7	181	13.9	81	44.8	
≥8	67	5.1	36	53.7	
Body pain in past 4 weeks					<0.01
None/very mild	763	58.5	256	33.6	
Mild	306	23.5	111	36.3	
Moderate/severe	236	18.1	118	50.0	
Level of satisfaction with physician					<0.01
Very	607	46.5	198	44.2	
Somewhat	510	39.1	204	40.0	
Not very or not at all	188	14.4	83	32.6	
Level of trust in physician					0.12
Completely or a lot	680	52.1	261	38.4	
Some	395	30.3	131	33.2	
Little or not at all	230	17.6	93	40.4	
Addiction to alcohol or drugs in the last year					0.16
No	1,286	98.5	475	36.9	
Yes	19	1.5	10	52.6	
Obesity in the last year					0.29
No	1,243	95.2	458	36.9	
Yes	62	4.8	27	43.6	

* Indicates the prevalence of CAM use among that subgroup of the population.

^† ^*p *value based on an unadjusted chi-square test of association.

**Figure 1:**
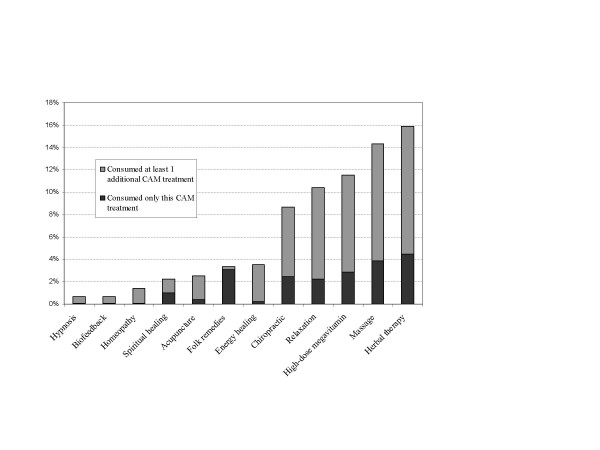
Percentage of complementary and alternative medicine (CAM) use among active-duty US Navy and Marine Corps personnel, from least used (hypnosis) to most used (herbal therapy). For each CAM treatment, the percentage of individuals who reported using at least one additional CAM treatment and the percentage of individuals who reported using only that CAM treatment are shown.

CAM use was reported in more than one third of study participants (37.2%). The least frequently used CAM treatments were hypnosis, biofeedback, and homeopathy, while the most frequently used treatments were herbal therapy, massage, and high-dose megavitamin therapy (See Figure 1). A person who reported using any one type of treatment was likely to report using others, although folk remedies were most often used alone rather than with other CAM treatments. Several treatments were consistently reported together; almost 55% of individuals who used acupuncture also used massage; 67% of individuals who used hypnosis also used relaxation, and 78% of individuals who used homeopathy also used herbal therapy.

Regression diagnostics for investigation of the pairwise correlations and the variance inflation factor suggested that no two variables were highly correlated and there was no discernible multicollinearity among the variables. There was no significant multiplicative interaction found between body pain and number of sick days. A manual backward elimination approach was used to investigate variable significance and confounding. Service branch, occupational category, marital status, and number of sick days were removed from the model because they were not significantly predictive of CAM use and did not display evidence suggesting possible confounding as measured by a 15% difference in odds ratio.

In the final model, women were more than twice as likely to report CAM use than men (OR = 2.18; 95% CI, 1.66–2.87) (Table 3). Officers were 50% more likely to report CAM use than were enlisted personnel (OR = 1.53; 95% CI, 1.15–2.04). Those who reported moderate to severe body pain were nearly twice as likely to report CAM use in comparison with those with none or very mild body pain (OR = 1.91; 95% CI, 1.39–2.61). Those who reported being "not very satisfied" with their conventional physician were almost 60% more likely to report CAM use when compared with those who reported they were "very satisfied" (95% CI, 1.11–2.28).

**Table 3:** Adjusted Odds Ratios and Confidence Intervals for Reported Complementary and Alternative Medicine Use in a Healthy US Military Population

Variable	Total n = 1,305	CAM Use n = 485	OR	95% CI
Gender				
Male^†^	1,009	338	1.00	--
Female	296	147	2.18	1.66, 2.87
Rank				
Enlisted^†^	1,009	356	1.00	--
Officer	296	129	1.53	1.15, 2.04
Race/ethnicity				
White^†^	871	355	1.00	--
Black	183	55	0.60	0.42, 0.86
Hispanic	119	29	0.44	0.28, 0.69
Asian/Pacific Islander	84	28	0.78	0.48, 1.28
Other	48	18	0.75	0.40, 1.39
Body pain in past 4 weeks				
None/very mild^†^	763	256	1.00	--
Mild	306	111	1.14	0.85, 1.52
Moderate/severe	236	118	1.91	1.39, 2.61
Level of satisfaction with physician				
Very^†^	607	198	1.00	--
Somewhat	510	204	1.44	1.11, 1.86
Not very or not at all	188	83	1.59	1.11, 2.28

* CAM indicates complementary and alternate medicine; OR, adjusted odds ratio; CI, confidence interval.

^†^Reference category.

### Survey reliability

To investigate reliability of responses in the initial survey, an abbreviated follow-up survey was mailed to a 33% random sample of the individuals who completed the first questionnaire. Of these, 146 (30.5%) completed the follow-up survey. On average, follow-up surveys were completed and returned within 15 months of initial surveys. The kappa statistic for measurement of concordance between the two surveys not due to chance was high for gender (κ = 0.90), race (κ = 0.87), household income (weighted κ = 0.62), and educational level (weighted κ = 0.79). The kappa statistics revealed moderate agreement for feeling of general health (weighted κ = 0.54), and fair agreement for both satisfaction with physicians (weighted κ = 0.30) and number of CAM treatments reported (weighted κ = 0.38) [28].

## Discussion and conclusion

As the use of complementary and alternative medicine becomes more widespread, it is important to understand potential effects on diverse medical and health care systems, practices, trends, and benefits. The DoD Force Health Protection program seeks to create a military force fully protected from preventable health threats throughout their military service. This includes assessing potential risks or benefits from unconventional medical alternatives and supplementation. For this reason, it is important to begin to understand prevalence of CAM use in active military personnel as well as general beliefs associated with CAM use in this population.

This report used responses to a postal questionnaire to compare the characteristics of military personnel who used one or more forms of CAM and personnel who did not use any of the 12 CAM treatments. Among the US Navy and Marine Corps personnel who participated in this study, 37% reported using at least one form of CAM in the past year. The two most commonly reported treatments were consistent with that of civilian reports. However, high-dose megavitamin use was the third most frequently reported CAM treatment (11%) and was not in the top four for civilian uses. This may reflect behaviors of a healthy population whose occupational requirements include health measures for advancement and who may believe their health to be positively impacted by the use of high-dose megavitamins.

Consistent with the literature, these data suggest that the odds of CAM use are significantly greater among women and White, non-Hispanic people after controlling for possible differences in other influential characteristics [1,15,30,31]. The finding that officers were more likely to report CAM use, independent of education and salary, has not been noted previously and may reflect a more career-conscious population whose focus on fitness and leadership may encourage physical or mental performance-enhancing supplementation.

Most interesting were the self-reported health questions concerning general health, number of sick days in the past year, body pain in the past 4 weeks, and level of satisfaction with physician. Compared with 9.0% of the general US population reporting poor or fair general health [32], in this study 4.4% of participants reported poor or fair general health. Among those who reported CAM use, 5.6% indicated their general health was poor or fair. Those having moderate to severe body pain were twice as likely to report CAM use independent of other factors including education and salary. Although this cross-sectional study design did not permit a temporal investigation of the finding, one might speculate that persons experiencing increased body pain may search for ways to alleviate the pain through complementary or alternative means. The finding that those who reported not being very satisfied with their physician were more likely to report CAM use is consistent with a population that seeks additional means to improve health if conventional care is unsatisfactory.

This study had a number of important limitations. These data were self-reported and may have been influenced by bias. With less than half of those contacted electing to participate, these data may not be representative of all Navy and Marine Corps personnel. Although the respondents' demographic characteristics represented the target population reasonably well, reasons for nonreponse were not obtained. Generalizing these data to all Navy and Marine Corps personnel should be done carefully. Additionally, these data do not represent the other services in the US military, Army and Air Force, which may be different with respect to CAM use. Additionally, these data do not capture the very heterogeneous nature of product quality or dose levels that may be used. Lastly, the results of the reliability analysis should be noted. Kappa statistics were near perfect for variables such as gender, race, and education, but fair to moderate for satisfaction with physician, feeling of general health, and overall number of CAM treatments reported. This may reflect a dynamic population with members trying new and different treatments during the average of 15 months between the first survey and the second survey. The relative variability of many of the answers to the questions included in the survey are dependent on multiple factors underlining a need for longitudinal analyses of CAM use. Other limitations to the Kappa statistic include being dependent on the true prevalence of the variable being examined, and lacking portability to other populations[33].

Despite its limitations, this study has a number of unique characteristics that add to our understanding of CAM use in healthy populations. Employing regression techniques allowed analysis of factors that influence the use of CAM while simultaneously adjusting for other influential factors. Although the study population was not extremely large, it permitted robust odds ratio estimates and considerable statistical power to detect many differences in characteristics based on CAM use. Additionally, with greater than 95% reporting good or better health, inferences could be made based on a healthy population.

In summary, approximately 37% of a healthy US Navy and Marine Corps population who participated in this postal questionnaire reported the use of at least one CAM treatment in the previous 12 months. This is consistent with the reported prevalence in the general population and should be of interest within the framework of the dietary and physical fitness regimen of the US military. While CAM therapies such as chiropractic care have been a part of the military health care system for over a decade, [34] it is important to understand CAM treatments so that guidelines may be created for personnel who choose to use any of the diverse methods of treatment. Future prospective studies using objective measures of health as well as measures of health related quality of life should attempt to identify health benefits or risks between conventional medicine and CAM.

## Competing interests

The author(s) declare that they have no competing interests.

## Authors' contributions

TS and BS performed the statistical analysis. All authors helped conceive the study, participated in its design and coordination, and helped to draft the manuscript. All authors read and approved the final manuscript.

## Pre-publication history

The pre-publication history for this paper can be accessed here:


